# Direct Synthesis of Zinc-Blende ZnSe Nanoplatelets

**DOI:** 10.1021/acsomega.4c02356

**Published:** 2024-06-12

**Authors:** Muhammed Said Es, Ebrar Colak, Aysenur Irfanoglu, Yusuf Kelestemur

**Affiliations:** Department of Metallurgical and Materials Engineering, Middle East Technical University, Ankara 06800, Turkey

## Abstract

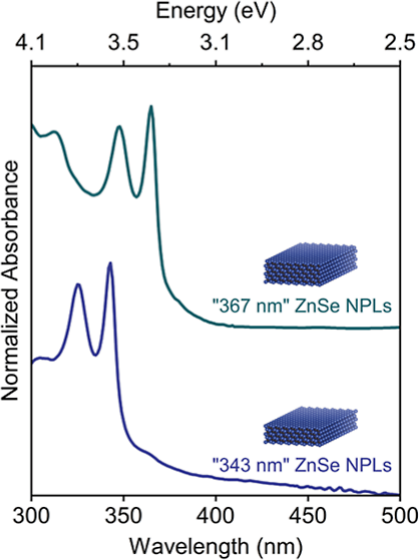

The distinct optical properties and electronic structures of two-dimensional colloidal nanoplatelets (NPLs) have garnered significant scientific and practical interest. However, concerns regarding the toxicity of cadmium-based NPLs and their limited spectral coverage show the importance of developing nontoxic alternatives. In this study, we devised a new synthetic approach for the direct synthesis of zinc-blende (ZB) ZnSe NPLs. By introducing two different zinc precursors, short-chain metal carboxylate- zinc acetate, and metal halide-zinc chloride, we successfully synthesized two-dimensional ZB ZnSe NPLs. By modifying the reaction parameters, we obtained two different populations of ZnSe NPLs, characterized by the first absorption peak at “343” and “367 nm”. Ostwald ripening experiments further confirmed the formation of 2D ZnSe NPLs by the observed discrete growth mechanism. Lastly, we investigated the impact of surface ligands on the excitonic properties of ZB ZnSe NPLs by treating their initially carboxylic acid-capped surface with oleylamine. Remarkably, we observed significant red-shifting in the first excitonic peaks, up to 130 meV, in a reversible manner, demonstrating further tunability of excitonic features in ZnSe NPLs. We anticipate that our findings will serve as a catalyst for further exploration of nontoxic two-dimensional materials, fostering their investigation and application in various fields.

## Introduction

Two-dimensional semiconductor nanoplatelets (NPLs), also referred to as colloidal quantum wells, have gained considerable attention with their distinct optical properties and electronic structures.^1^ These NPLs closely resemble epitaxially grown quantum wells, characterized by lateral dimensions on the order of tens of nanometers and thicknesses comprising several atomic layers.^2^ Owing to the strong quantum confinement effect along their magic sized vertical thicknesses, they exhibit thickness-dependent absorption and emission characteristics.^3^ Moreover, with the colloidal synthesis of NPLs having an atomically flat surface, colloidal NPLs exhibit suppressed inhomogeneous broadening, resulting in narrow emission line widths.^4^ Furthermore, these solution-processable NPLs possess strong absorption cross-section, giant oscillator strength, fast radiative lifetime, and suppressed Auger recombination.^5,6^ The exciting properties of these emerging two-dimensional NPLs make them highly desirable for optoelectronic applications spanning from light-emitting devices to lasers.^7−11^

In recent years, cadmium chalcogenide-based NPLs have emerged as some of the most extensively studied materials. Better understanding of their growth conditions has facilitated the control of their crystal structures during synthesis, enabling the reproducible synthesis of cubic zinc-blende (ZB)^3^ or hexagonal wurtzite (WZ) cadmium chalcogenide-based NPLs.^12^ Specifically, ZB cadmium chalcogenide-based NPLs have been synthesized with well-defined and adjustable vertical thicknesses. Moreover, their complex heterostructures including core/crown (laterally grown shell)^13−15^ and core/shell (vertically grown shell)^16−18^ have been developed with precise control over their chemical compositions such as alloyed core, alloyed shell, and gradient shell. The continuous progress in synthetic approaches of two-dimensional NPLs have led to the achievement of almost near-unity photoluminescence quantum yield (PLQY) and improved stability, reaching the performance of spherical-shaped colloidal nanocrystals.^19,20^ Nonetheless, several challenges persist with cadmium-containing colloidal NPLs. Concerns regarding the toxicity of cadmium limit the commercial use of these newly engineered nanocrystals.^21^ Additionally, the best-performing colloidal NPLs typically exhibit emission in the red spectral range, necessitating the development of nontoxic two-dimensional NPLs that cover the blue and green spectral range.

At this point, zinc chalcogenide-based semiconductors have been considered as a promising alternative with their suitable energy band gaps.^22,23^ Particularly, ZnSe having a direct band gap of 2.7 eV^24^ is very attractive for blue light-emitting applications. So far, efforts have predominantly focused on spherical-shaped ZnSe-based NCs,^25^ with less exploration into other morphologies such as nanowires (NWs),^26^ nanorods (NRs),^27^ and NPLs.^28−30^ In addition to the bare ZnSe NCs of varying sizes, studies have also investigated alloying high band gap ZnSe with low band gap ZnTe to further engineer emission colors within the blue/green spectral range (420–500 nm).^31^ Recently, Kim et al. demonstrated that the PLQY of ZnSe-based NCs could be increased to nearly unity by surface treatment with hydrofluoric acid to eliminate trap sites and with zinc chloride to effectively passivate the surfaces.^32^ These highly efficient blue-emitting core/shell ZnSe-based NCs were successfully integrated into light-emitting devices, achieving an impressive external quantum efficiency of 20.2%.^32^

Despite the significant advancements made with spherical-shaped ZnSe-based NCs, exploring the two-dimensional form of semiconductor NCs presents promising opportunities for achieving spectrally pure emission, enhancing absorption cross sections, accelerating radiative recombination rates, and suppressing Auger recombination.^6^ With this motivation, two-dimensional forms of ZnSe nanocrystals have been also explored. The first examples of two-dimensional ZnSe NPLs are synthesized by using a mixture of short-chain and long-chain alkylamine as a solvent/ligand, revealing a hexagonal WZ crystal structure with sharp absorption peaks.^28^ However, the first absorption peak of these WZ NPLs is generally located around 347 nm and suffers from the thickness tunability. Pang et al. reported that the synthesis of thicker ZnSe NPLs having WZ crystal structure is restricted by the requirement of large formation energy, and it is challenging to tune their thickness.^33^ The study presented by Cunningham et al.^34^ further supported these findings. They showed that WZ ZnSe NPLs having an absorption onset at 345 nm was converted into thicker ZnSe NPLs having an absorption onset at 380 nm through the Ostwald ripening process under prolonged heating. However, the resulting ZnSe NPLs did not preserve their initial wurtzite crystal structure and exhibit a ZB crystal structure. This is the first report in the literature showing the possibility of obtaining ZB ZnSe NPLs via a colloidal approach, thus, inspiring further exploration into direct synthesis pathways. Also, the ease of thickness tunability in cadmium chalcogenide-based NPLs with ZB crystal structures increases the interest for the development of ZnSe NPLs with ZB crystal structures.^35^ Thus, the quest for a novel synthetic approach to directly produce 2D ZB ZnSe NPLs persists.

In this study, we demonstrate the direct synthesis of ZnSe NPLs featuring a ZB crystal structure by modifying a traditionally used recipe for the synthesis of spherically shaped ZnSe NCs. By incorporating two distinct zinc precursors, short-chain metal carboxylates—zinc acetate and metal halides—zinc chloride, we altered the growth kinetic and changed the surface energy. These modifications facilitated the direct synthesis of two-dimensional ZnSe NPLs having a ZB crystal structure, characterized by the first absorption peak at “343 nm”.^34^ Furthermore, we explored the potential for thickness adjustability in ZnSe NPLs through Ostwald ripening experiments. We observed a discrete growth of ZnSe NPLs during Ostwald ripening experiments and obtained a thicker population of ZnSe NPLs having the first absorption peak at “367 nm”. We also demonstrate that this thicker population of ZnSe NPLs can be synthesized directly by modifying the synthesis conditions. Lastly, we show that by treating the surface of initially carboxylic acid-capped ZnSe NPLs with oleylamine, the excitonic properties of ZnSe NPLs can be further engineered in a reversible manner.

## Experimental Section

### Chemicals and Materials

Zinc stearate (Zn(St)_2_, purum, 10–12% Zn basis, product code: 102416828, Sigma-Aldrich), zinc acetate dihydrate (Zn(Ac)_2_·2H_2_O, >99.0%, product code: 102423818, Sigma-Aldrich), zinc chloride (ZnCl_2_, >98%, product code: S42826-20208B13, Fluka), selenium (Se, trace metal basis >%99.5, 100 mesh, product code: 209651-250G, Aldrich), octadecene (ODE, technical grade 90%, product code: O806-1L, Sigma-Aldrich), and oleic acid (OA, technical grade 90%, product code: 364525-1L, Sigma-Aldrich) were used. Hexane (96%, extra pure) and ethanol (99%) were purchased from ISOLAB and used without purification.

### Synthesis of ZnSe NCs

A typical synthesis procedure for ZnSe NCs is similar to previously published reports.^31^ Initially, a mixture containing 0.5 mmol ZnSt_2_, 0.25 mmol Se, and 10 mL of ODE was placed into a 50 mL three-necked flask and degassed at 100 °C for 30 min. After degassing, the temperature was increased to 250 °C under N_2_ gas and maintained at that temperature for 1 h. Then, the reaction mixture was cooled to room temperature. The resulting mixture was then transferred to a centrifuge tube by adding 1 mL of *n*-hexane. After centrifugation at 4000 rpm for 10 min, the precipitated NCs were dissolved in 6 mL of *n*-hexane and centrifuged again. Finally, the supernatant solution was precipitated using 4 mL of ethanol to purify ZnSe nanocrystals. The final precipitate was dissolved in 6 mL of *n*-hexane.

### Synthesis of ZnSe NPLs "343 nm"

A procedure for synthesizing ZnSe NPLs involves modifications from the typical ZnSe NC synthesis. For a typical synthesis, 0.5 mmol ZnSt_2_, 0.25 mmol Se, and 10 mL of ODE were loaded to a 50 mL three-necked flask, which is then degassed at 100 °C for 30 min. Subsequently, the reaction temperature was increased to 220 °C under N_2_ gas. When the temperature reached 150 °C, 0.45 mmol zinc acetate dihydrate and 0.15 mmol ZnCl_2_ were swiftly added to the reaction mixture. The reaction mixture was kept at 220 °C for the growth of ZnSe NPLs. During the reaction, several aliquots were taken in order to monitor the formation of NPLs. After 1 h, the resulting reaction mixture was cooled to room temperature by air blowing, and 2 mL of OA was added to the reaction mixture when temperature was decreased to 150 °C. The reaction mixture was then transferred to a centrifuge tube by adding 1 mL of *n*-hexane and centrifuged for 10 min at 4000 rpm. The precipitated NPLs were dissolved in 6 mL of *n*-hexane and subjected to an additional 10 min of centrifugation at 4000 rpm. The supernatant solution obtained after centrifugation was used for further characterization.

### Ostwald Ripening Experiment

To observe Ostwald ripening in 343 nm ZnSe NPLs, the established synthesis method was employed with small modifications. First, ZnSt_2_ (0.5 mmol), Se (0.25 mmol), and 10 mL of ODE were loaded in a 50 mL three-necked flask and degassed at 100 **°**C for 30 min. Then, the reaction temperature was increased to 240 °C under N_2_ gas and previously weighed zinc acetate dihydrate (0.45 mmol) and ZnCl_2_ (0.15 mmol) were added to the reaction at 150 °C. During the reaction, several aliquots were taken in order to check the formation of NPLs and Ostwald ripening phenomena. After 8 h of prolonged heating at 240 °C, the reaction mixture was cooled to room temperature. During the cooling, 2 mL of oleic acid (OA) was added to reaction mixture at 150 °C. The reaction mixture was then transferred to a centrifuge tube, and 1 mL of *n*-hexane was added and centrifuged for 10 min at 4000 rpm. The resulting precipitated NPLs were dissolved by the addition of 6 mL of *n*-hexane and subjected to an additional 10 min of centrifugation. Subsequently, the supernatant was collected for additional characterization.

### Synthesis of ZnSe NPLs "367 nm"

The synthesis procedure for “367 nm” ZnSe NPLs typically proceeds as follows: Initially, ZnSt_2_ (0.5 mmol), Se (0.25 mmol), and 10 mL of ODE were introduced into a 50 mL three-necked flask and degassed at 100 °C for 30 min. After the mixture was degassed, the reaction temperature was set to 260 °C under N_2_ gas. At 200 °C, previously weighed zinc acetate dihydrate was added into the reaction in powder form. Then, when temperature was reached to 205 °C, 0.15 mL of ZnCl_2_ dissolved in water (1 M) was swiftly injected to the reaction. During the reaction, several aliquots were taken in order to check the formation of NPLs. After 1 h, the reaction mixture was cooled to room temperature. During cooling, 2 mL of OA was added to the reaction at 150 °C. ZnSe NPLs having a population of 367 nm were purified in a similar way.

### Characterization

Absorption and photoluminescence (PL) spectra of colloidal NPLs were collected with an Edinburgh Instruments FS5 spectrofluorometer. X-ray diffraction patterns were recorded by using a Bruker D8 Advance diffractometer with Cu K_α_ radiation. Transmission electron microscopy (TEM) images were acquired by using a JEOL JEM 2100F HRTEM microscope operating at 200 kV. Fourier-transform infrared spectroscopy (FTIR) measurements were conducted using a Shimadzu IRTracer-100 device configured in an attenuated total reflectance setup by drop-casting samples onto the diamond cell.

## Results and Discussion

In this study, we investigated the direct synthesis of ZnSe NPLs having a ZB crystal structure. As a starting point, we focused on choosing an appropriate recipe that could be modified for the synthesis of 2D ZnSe NPLs. We specifically targeted recipes known to produce ZnSe NCs with a ZB crystal structure, while excluding those employing diethylzinc as a zinc precursor and trioctylphosphine–selenium (TOP–Se) as a selenium precursor.^25^ In addition to the raised concern about their handling, the high reactivity of these precursors makes it difficult to control the reaction kinetics. Thus, we decided to choose a recipe including zinc stearate as a zinc precursor, elemental selenium as a selenium precursor, and octadecene (ODE) as a solvent.^31^ The similarity of the chemicals used in this recipe to those employed in the synthesis of two-dimensional cadmium chalcogenide-based NPLs further encouraged us to proceed with this protocol.^2^

**Figure 1:**
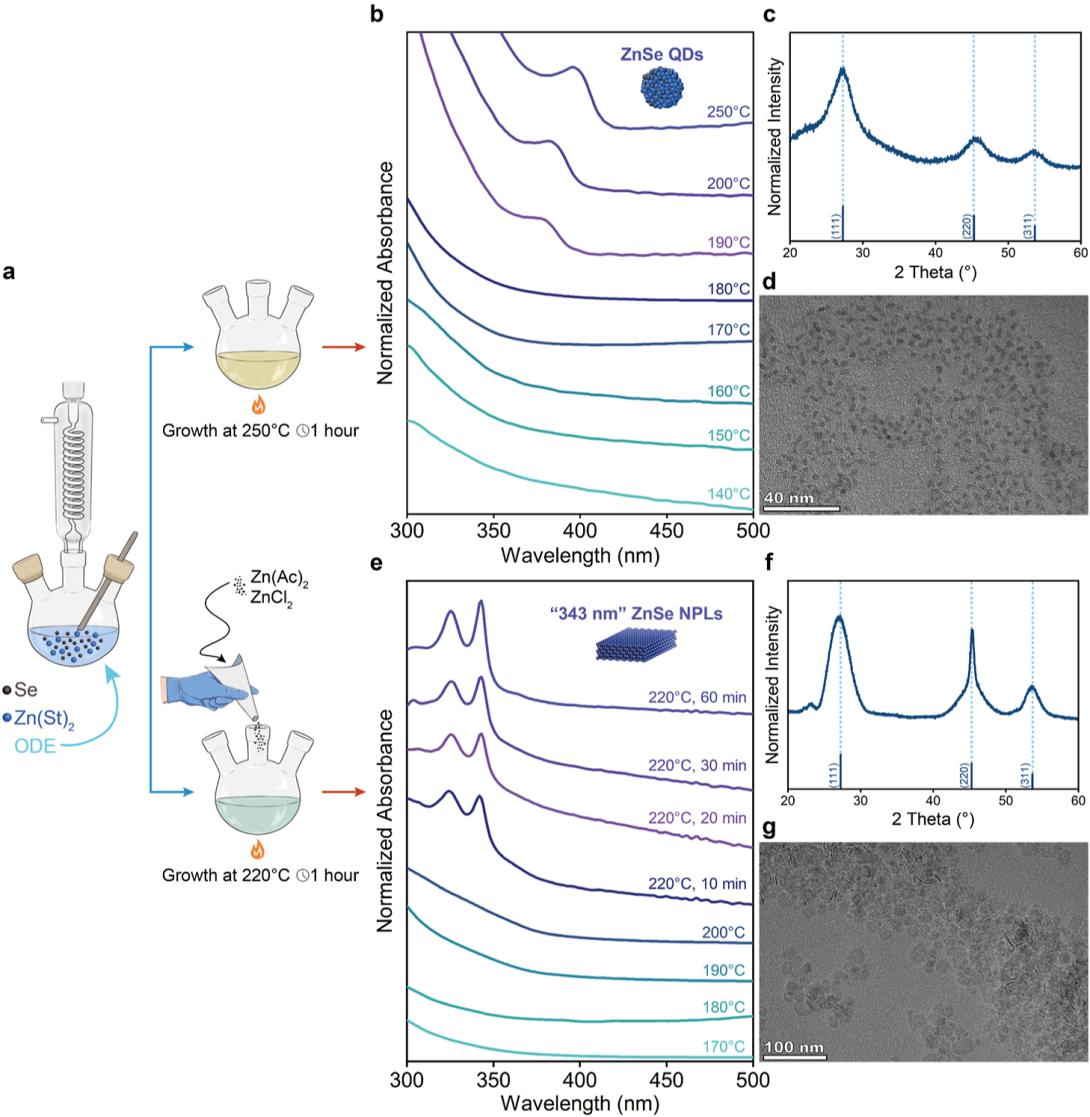
(a) Schematics for our developed synthetic approach yielding spherical-shaped NCs and two-dimensional NPLs. (b) Normalized absorption spectra of ZnSe NCs taken at different temperatures during heating and prolonged growth. (c) X-ray diffraction (XRD) pattern of spherical-shaped ZnSe NCs. (d) TEM image of spherical-shaped ZnSe NCs. (e) Normalized absorption spectra of ZnSe NPLs having first absorption peak located at “343 nm”. (f) Diffraction pattern of two-dimensional ZnSe NPLs, and their corresponding TEM image (g).

First, we synthesized ZnSe NCs by using a heat-up synthesis protocol. After the degassing step, the reaction mixture was heated to 250 °C for the growth of nanocrystals. To observe the nucleation and growth stages, we took several aliquots at different temperatures during heating. The absorption spectra of taken aliquots are depicted in Figure 1b. The nucleation of nanocrystals starts around 190 °C, evidenced by the appearance of a shoulder around 380 nm in the absorption curve. As the temperature increased, the excitonic peak of ZnSe NCs was shifted to longer wavelengths and became more pronounced, showing the growth of ZnSe NCs. The diffraction pattern of the synthesized ZnSe NCs is presented in Figure 1c, and it confirms their ZB crystal structures. The intensities of diffraction peaks were found to be similar to the bulk form of ZB ZnSe, suggesting the formation of isotropic shaped NCs. We further analyzed the shape of the synthesized ZnSe NCs by using TEM, and it was shown that our followed synthesis protocol yields a spherical-shaped ZnSe NCs with a diameter of 2.46 ± 0.41 nm.

Then, we focused on adapting this synthetic approach for the synthesis of two-dimensional NPLs. Initially, we tried the incorporation of short-chain metal carboxylates to the synthesis, inspired by the synthesis of cadmium chalcogenide-based NPLs.^2^ Experimental observations, together with the model proposed by Riedinger et al., suggest that the introduction of short-chain metal carboxylates induces growth instability and promotes the anisotropic growth of nanocrystals.^36^ The limited solubility of short-chain metal carboxylates in ODE leads to the formation of the localized supersaturated region within the reaction mixture, thereby favoring anisotropic growth through alterations in growth kinetics—specifically, surface reaction-limited growth.^37^ Thus, we investigated the addition of zinc acetate dihydrate in a powder form to the reaction mixture at various temperatures and concentrations. However, throughout these trials, we did not observe the sharp excitonic features in the absorption spectra expected from two-dimensional NPLs (Supporting Information S1a).

In the study of Riedinger et al., they also discuss the limitation imposed on the growth of two-dimensional NPLs by an energy barrier.^36^ For instance, the direct synthesis of CdSe NPLs with thicknesses of 3, 4, and 5 monolayers (MLs) is achievable with the addition of short-chain metal carboxylates. However, the synthesis of thicker NPLs is impeded by the energy barrier. Recent investigations have demonstrated that the addition of metal halides can overcome this energy barrier, enabling the direct synthesis of thicker CdSe NPLs by incorporating cadmium chlorides.^35,38^ Consequently, we explored the addition of metal halide salts to modify the surface energy. Upon adding ZnCl_2_ powder to the reaction mixture, we observed the emergence of sharp excitonic features in the absorption spectra (Supporting Information, Figure S1b). These sharp features are typically attributed to the separation of light-hole and heavy-hole transitions in two-dimensional NPLs. Nonetheless, the relative intensities of these excitonic features differed from those observed in cadmium chalcogenide-based NPLs. This discrepancy in excitonic peaks could stem from either incomplete growth of ZnSe NPLs or strain relaxation induced by metal halides.^39^ Therefore, we redirected our focus toward further investigating synthesis conditions by adjusting the type and amount of the injected precursor.

Upon incorporation of a mixture of zinc acetate dihydrate and zinc chloride powders, we observed more pronounced sharp absorption peaks. In a typical synthesis, these mixed zinc precursors were introduced in powder form before the nucleation of ZnSe NCs around 150 °C, followed by growth at 220 °C. Figure 1e illustrates the absorption spectra from a typical synthesis. The injection of additional zinc precursors led to the appearance of two distinct sharp excitonic peaks in the absorption spectra. The first excitonic peak at 343 nm was attributed to the heavy-hole transition, while the second excitonic peak at 327 nm was associated with the light-hole transition, characteristic of two-dimensional NPLs. With continued growth at this temperature, the first excitonic peak at 343 nm remained in the same spectral position but exhibited an increased intensity, indicating continuous growth of NPLs. It is also important to note that when we limited the growth time to 30 min, we obtained similar intensity levels of absorption peaks with the synthesis performed only by injecting ZnCl_2_ (Supporting Information, Figure S2). This finding suggests that this discrepancy in absorption may be attributed to incomplete growth of ZnSe NPLs. We also measured the PL spectra of the synthesized ZnSe NPLs; however, we did not observe any band-edge emission.

We also confirmed the growth of 2D ZnSe NPLs through structural characterization. TEM images revealed the growth of these NPLs, with some standing on their large lateral surfaces, while others were stacked within each other, a common observation in two-dimensional NPLs. The thickness of ZnSe NPLs having a population of “343 nm” was determined by measuring the thickness of several NPLs that were stacked within each other, yielding a value of 1.14 ± 0.25 nm. Notably, the synthesized ZnSe NPLs exhibited nonuniform size distribution in lateral dimensions, and their edges were not as sharp as those of CdSe NPLs.

The diffraction patterns of synthesized and purified 2D ZnSe NPLs are also depicted in Figure 1f. According to the observed diffraction peaks, synthesized ZnSe NPLs exhibit ZB crystal structure. Additionally, two distinct features were observed in the diffraction pattern of ZnSe NPLs, commonly associated with the formation of two-dimensional ZB NPLs.^40^ First, we observed that the intensities of diffraction peaks were different from those of the bulk form of ZB ZnSe. The increase in the intensity of the (220) peaks suggests the anisotropic growth of the synthesized nanocrystals. Second, splitting of (220) peaks into sharp and broad features was observed, also commonly seen in core-only CdSe NPLs. Also, ligands attached to the surface of NPLs induce strain formation, leading to distortion of the cubic unit cell. As a result, the unit cell shifts from cubic to tetragonal, as evidenced by the shifted peak positions of narrow and broad features in the (220) peak.^40^ These distinct features further validate the growth of anisotropic two-dimensional ZnSe NPLs with a ZB crystal structure.

We also investigated the growth direction of ZB ZnSe NPLs by utilizing high-resolution TEM (HRTEM) imaging. The fast Fourier transform (FFT) image obtained from a selective area on the wide facet of the ZnSe NPLs revealed that the zone axis could be identified as the [110] direction for our ZB ZnSe NPLs (Supporting Information, Figure S7). The angular relationship between the diffraction spots and the absence of four-fold rotational symmetry further validated that the wide facets of ZB ZnSe NPLs belong to {110} planes, consistent with the findings reported by Cunningham et al.^34^ These results stand in contrast to those of commonly studied ZB CdSe NPLs, where the wide facets are terminated with {100} planes, adding an intriguing dimension to our observations.^41^

In addition, we conducted Ostwald ripening experiments to explore the possibility of obtaining thicker ZnSe NPLs.^36^ Unlike in the case of spherical-shaped NCs where Ostwald ripening involves smaller nanocrystals dissolving while larger ones grow, the process occurs differently for two-dimensional NPLs. In NPLs, Ostwald ripening follows a discrete growth pathway, sequentially jumping in thickness from thinner NPLs to thicker ones.^37^ In our Ostwald ripening experiment, we used our developed recipe for synthesizing ZnSe NPLs with a population peak at 343 nm with minor modifications. We increased the growth temperature to 240 °C and maintained the reaction at that temperature for an extended period of time. During the prolonged heating, we took several aliquots at different time intervals for analysis.

**Figure 2:**
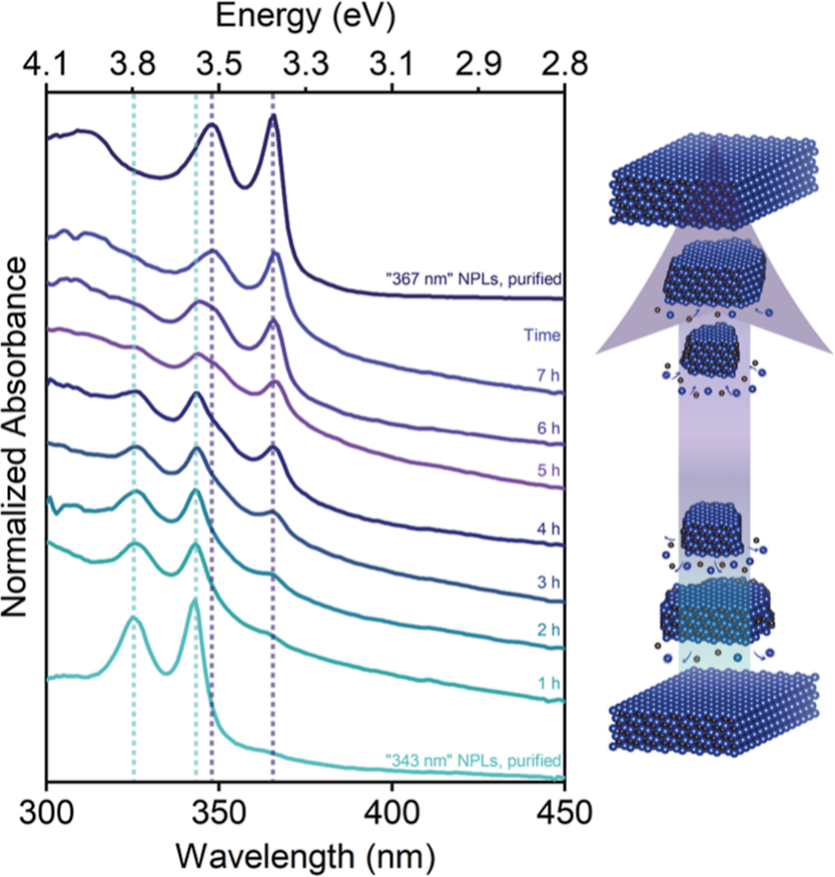
Ostwald ripening experiments performed with ZnSe NPLs having a population of “343 nm”. The absorbance spectra of aliquots taken during the Ostwald ripening experiments at prolonged heating, along with the schematic providing a visual representation of the changes occurring in the nanoplatelets’ size distribution, drawn based on the analysis of the absorption spectra.

The absorption spectra of these aliquots are presented in Figure 2. After 1 h of growth, we observed the formation of ZnSe NPLs with a population of “343 nm”. With further annealing at 240 °C, a new peak emerged around 367 nm, and it became more pronounced, indicating the growth of a thicker population of ZnSe NPLs. Concurrently, the excitonic peaks of ZnSe NPLs with a population of “343 nm” remained nearly unchanged in wavelength with slight blue-shifting (see Supporting Information, Figure S3). However, the intensity of these peaks gradually diminished and eventually disappeared after 7 h of annealing. The disappearance of excitonic peaks with slight blue-shifting suggests the dissolution of ZnSe NPLs with a population of 343 nm in lateral dimensions. If these NPLs were converted to thicker ones, we would expect a spectral shift in the excitonic features. On the other hand, the excitonic peaks of ZnSe NPLs, with a population of “367 nm”, exhibited minimal shifts in the position, displaying a slight red-shift, while their intensity increased during prolonged heating. These observations imply lateral growth of the ZnSe NPLs having a population of 367 nm over time. In conclusion, Ostwald ripening experiments further validate the formation of two-dimensional ZnSe NPLs by observing discrete growth characteristics, and it also shows the tunability of thickness in ZB ZnSe NPLs.

**Figure 3:**
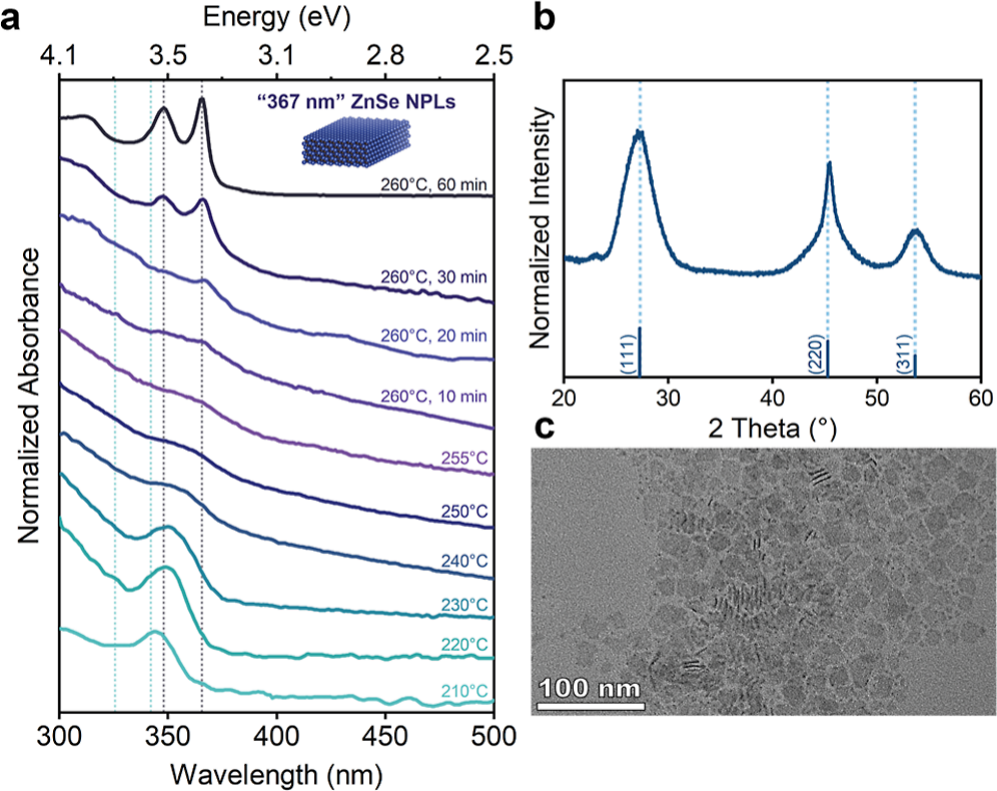
Optical and structural characterization of ZB ZnSe NPLs having a population of “367 nm”. Absorbance spectra (a), X-ray diffraction pattern (b), and TEM image of synthesized ZnSe NPLs (c).

Following the observation of thicker ZnSe NPLs with a population of 367 nm in the Ostwald ripening experiments, we investigated to modify our recipe to directly synthesize this new population. Studies on cadmium chalcogenide-based NPLs have indicated that increasing the growth temperature and delaying the injection of additional precursors promote the formation of thicker NPLs.^2^ Both strategies aim to reduce the amount of seeds in the reaction mixture, which could potentially convert into thinner populations of NPLs.^37^ Therefore, differing from our previous recipe, we raised the growth temperature to 260 °C and introduced the additional zinc precursors at a later stage, after the nucleation of ZnSe NCs around 200 °C. Figure 3a illustrates the absorption spectra from a typical synthesis. Following 20 min of growth at 260 °C, sharp excitonic features emerged, with the first absorption peak centered at 367 nm. With prolonged growth at this temperature, this peak intensified while remaining at the same spectral position. The absence of sharp excitonic features corresponding to ZnSe NPLs with a population of “343 nm” confirmed the direct synthesis of ZnSe NPLs with a population of “367 nm”, without Ostwald ripening phenomena. Structural characterization of this new population was also performed (Figure 3b). The diffraction pattern and TEM image confirmed the successful growth of ZB ZnSe NPLs with a population of “367 nm”. The thickness of the synthesized ZnSe NPLs was determined by measuring the thickness of several NPLs and was found as 1.46 ± 0.22 nm.

For comparison with our results on ZB ZnSe NPLs, we identified only one relevant report in the literature, published by the Talapin group.^34^ In this study, they presented the first example of ZB ZnSe NPLs with an absorption onset at “380 nm”, synthesized through the conversion from wurtzite (WZ) ZnSe NPLs with an absorption onset at “345 nm”. When we compared our directly synthesized ZB ZnSe NPLs, having populations of “343 nm” and “367 nm”, significant differences were noted in the first excitonic peaks. One possible explanation for these observed differences in the excitonic peaks could be attributed to variations in the thickness of the synthesized ZB ZnSe NPLs in these studies. Our ZnSe NPLs with a population of “367 nm” may correspond to an intermediate thickness between the populations with absorption onset at “343 nm” and “380 nm”. Another potential explanation may be related to the ligands attached to the surface of the NPLs. Previous studies have highlighted that unlike spherical-shaped NCs, any surface modification in colloidal NPLs results in significant shifts in the absorption peaks.^39,40,42−45^ For example, Antanovich et al. reported that ligand exchange of native oleic acid with aliphatic thiol or phosphonic acid on the surface of ZB CdSe NPLs induces a substantial shift of exciton transition energy of up to 240 meV.^40^ Similarly, Dirol et al. demonstrated that treating CdSe NPLs with halide salts causes a significant bathochromic shift in the absorption resonances, leading to a red-shift in the optical spectrum.^42,43^ These studies attributed the observed large shifts in absorption peaks to relaxation of quantum confinement and anisotropic strain in the crystalline structure. Additionally, the study conducted by Zhou et al.^45^ revealed that surface treatment of initially octylamine-passivated CdSe quantum belts with cadmium oleate led to a notable shift in the excitonic peaks. The process of ligand exchange in CdSe quantum belts introduced an additional layer of cadmium on their surface, thereby increasing their effective thickness. Consequently, together with the change in strain states, the increase in the effective thickness of CdSe quantum belts facilitated relaxation of quantum confinement, leading to the significant shift observed in excitonic peaks.

**Figure 4:**
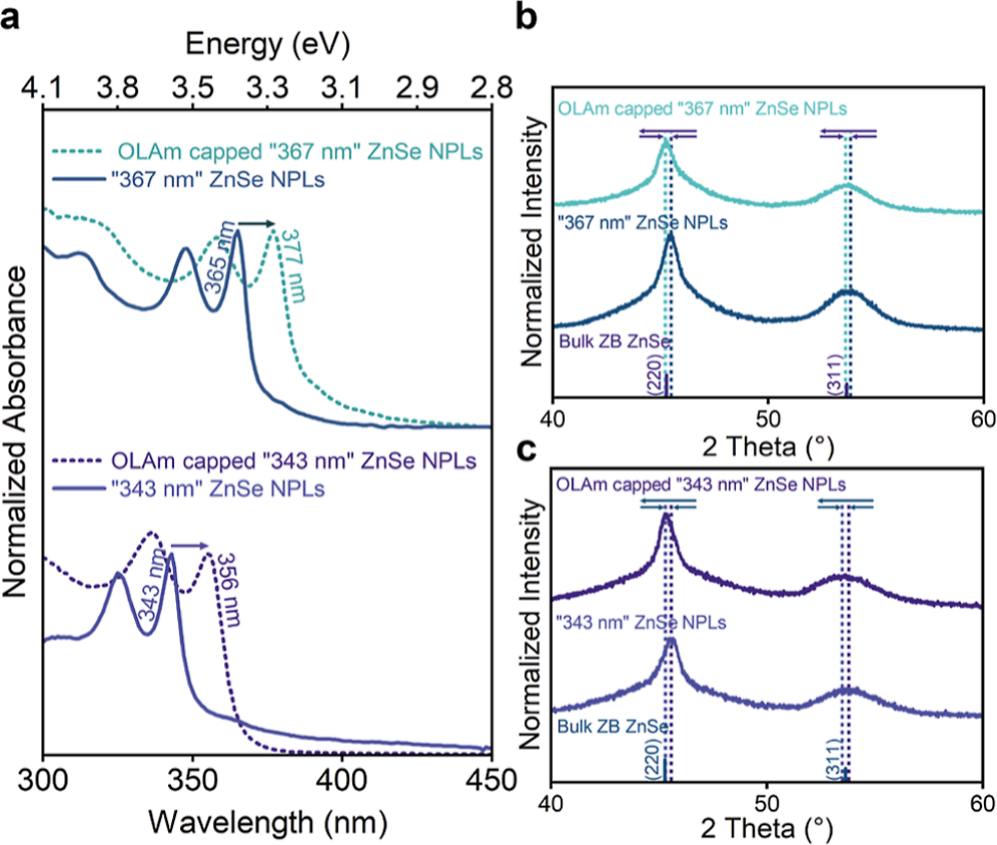
Absorption spectra of ZnSe NPLs having a population of “343 nm” and “367 nm” before and after the surface modification (a). Diffraction patterns of ZB ZnSe NPLs measured before and after the surface modification for population of “367 nm” (b) and “343 nm” (c).

Upon reviewing the synthesis protocol used by Cunningham et al.,^34^ we found that they employed a mixture of oleylamine and octylamine as solvents and ligands, which differs from our synthesis protocol. Therefore, we decided to investigate the surface properties of our ZnSe NPLs further, including the ligands attached to their surfaces, to better understand and potentially explain these differences in excitonic peaks. We studied the effect of oleylamine on the surface of our synthesized ZB ZnSe NPLs initially capped with carboxylic acid. The surfaces of ZB ZnSe NPLs were examined using infrared spectroscopy (IR) (Supporting Information, Figure S8). The absence of characteristic features associated with carboxylic acids, along with the emergence of peaks corresponding to oleylamine subsequent to the ligand exchange, indicates the successful completion of the ligand exchange process. The absorption spectra of ZB ZnSe NPLs with different surface motifs are depicted in Figure 4a. After treating the surface of ZnSe NPLs with oleylamine, we observed a shift in the first excitonic peaks to longer wavelengths in both populations of ZnSe NPLs albeit with different magnitudes. While the amount of shifting for thinner ZnSe NPLs was calculated as around 130 meV, it was found to be around 90 meV for thicker ZnSe NPLs.

For the case of ZB ZnSe NPLs with a population at “367 nm”, we observed that after treating the surface of the NPLs with oleylamine, the first excitonic peaks were shifted to longer wavelengths (377 nm), overlapping with the findings of the Talapin group study.^34^ The observed slight differences in excitonic peaks between ZnSe NPLs obtained through these two distinct approaches may be attributed to variations in the lateral dimensions of the synthesized NPLs and their different surface configuration.

To understand the origin of this large shift in the excitonic peaks of ZnSe NPLs, we analyzed the diffraction pattern of the ZB ZnSe NPLs before and after surface modification. The diffraction pattern revealed a change in the positions of the diffraction peaks following the surface modification. Upon detailed analysis of the (220) plane, which displayed splitting (Supporting Information, Figure S9), we observed an increase in the lattice parameters of the initial tetragonal unit cell with varying degrees in different directions. Hence, the change in the strain state of ZB ZnSe NPLs following ligand exchange could be considered as one factor contributing to the observed shift in excitonic peaks, aligning with previous studies. Moreover, the increase in thickness of the ZB ZnSe NPLs, coupled with distortion in the unit cell, facilitated the relaxation of quantum confinement, further influencing the shift in excitonic peaks. Additionally, enhanced exciton delocalization to the passivating ligand layers after ligand exchange might contribute to this shift. However, comprehensive systematic studies are required to precisely determine the individual contributions of each factor.

## Conclusions

In conclusion, we have devised a novel synthetic method for the direct synthesis of ZB ZnSe NPLs with two distinct populations: “343 nm” and “367 nm”. Through the proper selection of precursors and modulation of growth kinetics and surface energy, we demonstrated the possibility of synthesizing 2D NPLs with colloidal routes. Our findings are correlated with the previously described model for explaining the anisotropic growth of NPLs by Riedinger et al. and emphasize the significance of overwhelming energy barriers in the synthesis of two-dimensional NPLs. We believe that our study will inspire further exploration of nontoxic two-dimensional materials and pave the way for their investigation and application.

## Supporting Information Available

Characterization of ZnSe NPLs synthesized by only introducing Zn(Ac)_2_ and only introducing ZnCl_2_; optical characterization of ZnSe NPLs that synthesized for 30 min; TEM images for ZnSe NPLs having a population of “343 nm” and “367 nm”; FTIR spectra of the ZnSe NPLs before and after the ligand exchange; and detailed analysis of XRD patterns of ZnSe NPLs before and after the surface modifications (PDF)
