# Supplementary material for: Direct Synthesis of Zinc-Blende ZnSe Nanoplatelets
Source: ACS Omega. 2024 Jun 12;9(25):27438–45. doi: 10.1021/acsomega.4c02356 (PMC11209924; doi:10.1021/acsomega.4c02356)
Supplement: Supplementary file 1 — ao4c02356_si_001.pdf [file ao4c02356_si_001.pdf]

*Supporting Information*

# Direct Synthesis of Zinc Blende ZnSe Nanoplatelets

*Muhammed Said Es,[1] Ebrar Colak,[1] Aysenur Irfanoglu,[1] Yusuf Kelestemur[1*]*

*[1]Department of Metallurgical and Materials Engineering, Middle East Technical University, 06800, Ankara, Türkiye*

[*]Corresponding Author: yusufk@metu.edu.tr

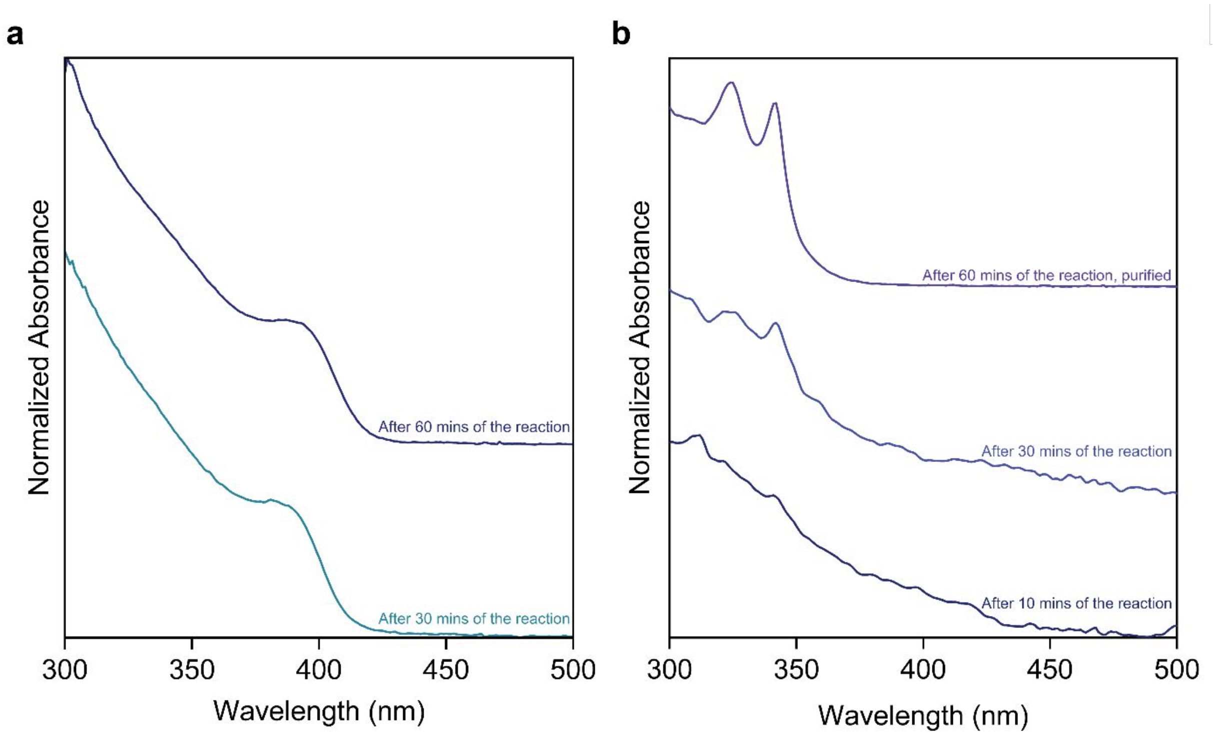


**Figure S1.** Absorption spectra of ZnSe NPLs synthesized by introducing (a) only $Zn(Ac)_2 .2H_2O$, and (b) only $ZnCl_2$.

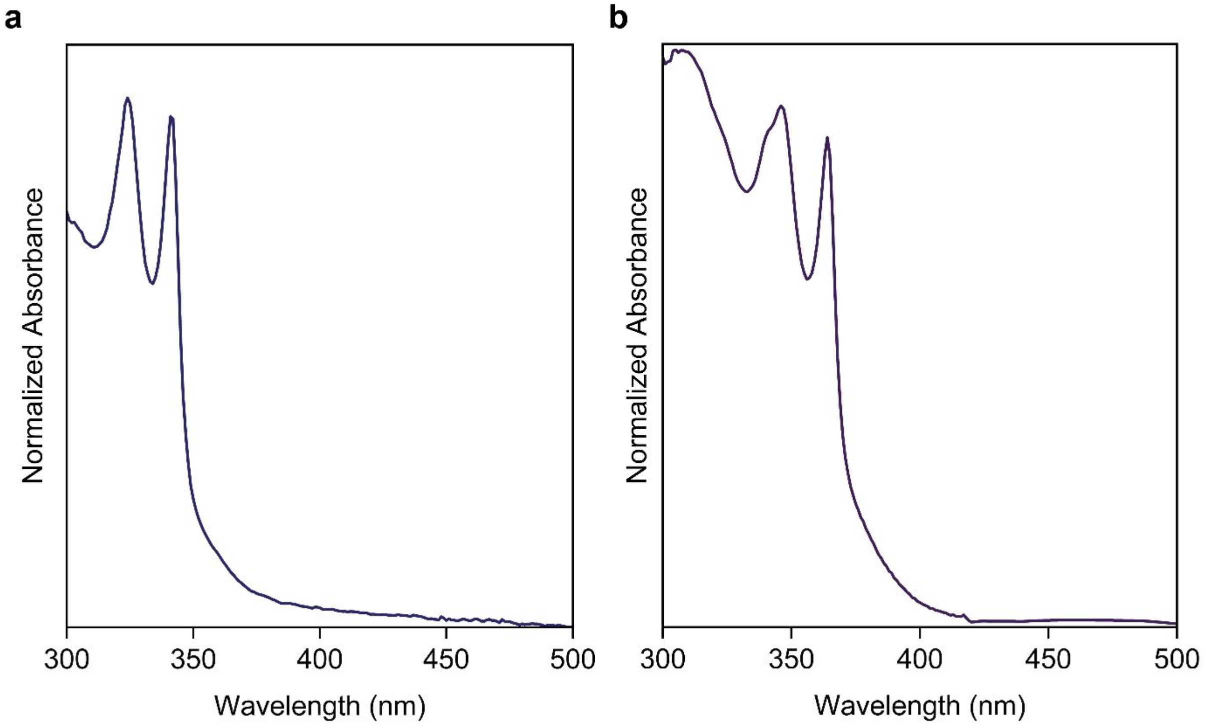


**Figure S2.** Absorption spectra of ZnSe NPLs having a population of (a) "343 nm" and (b) "367 nm" after 30 minutes of the reaction.

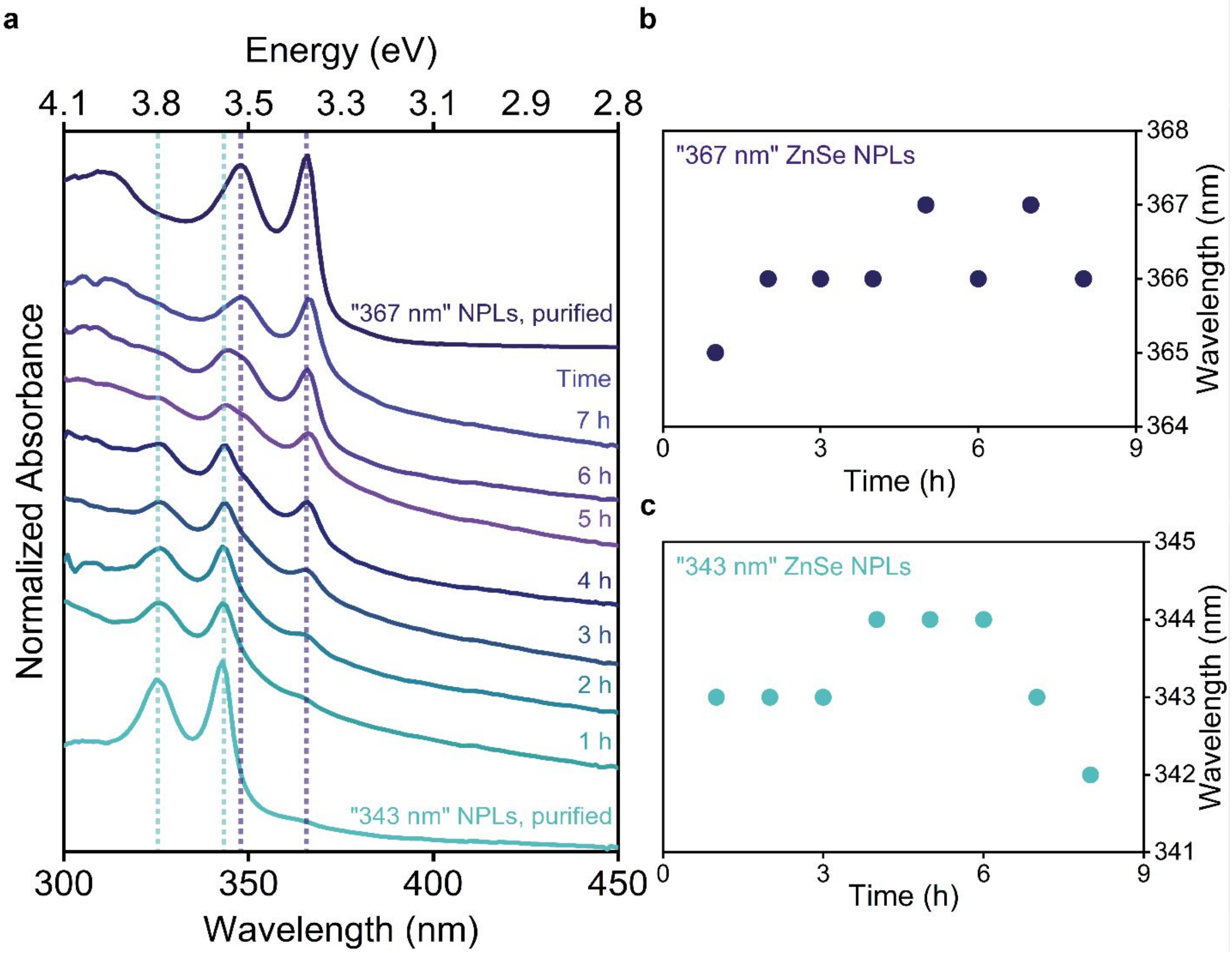


**Figure S3.** Ostwald ripening experiments were performed with ZnSe NPLs having a population of "343 nm". (a) The absorption spectra of aliquots taken at different time intervals during the prolonged heating. The shifting in the first excitonic peaks of ZnSe NPLs having a population of "367 nm" (b) and "343 nm" NPLs (c) as a function of time. The first excitonic peaks were determined from the corresponding absorption spectra by taking the second derivative.

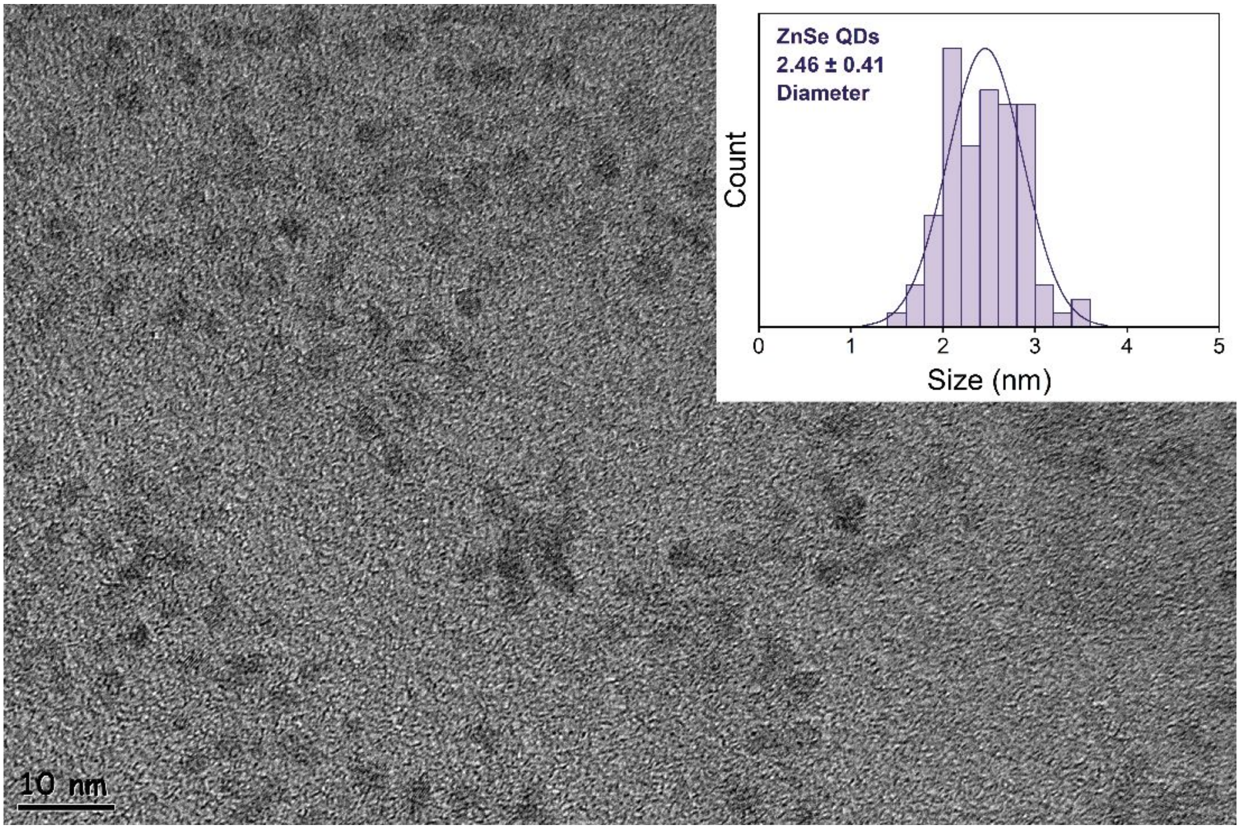


**Figure S4.** Transmission electron microscopy (TEM) image of spherical shaped ZnSe NCs together with the size distribution histogram.

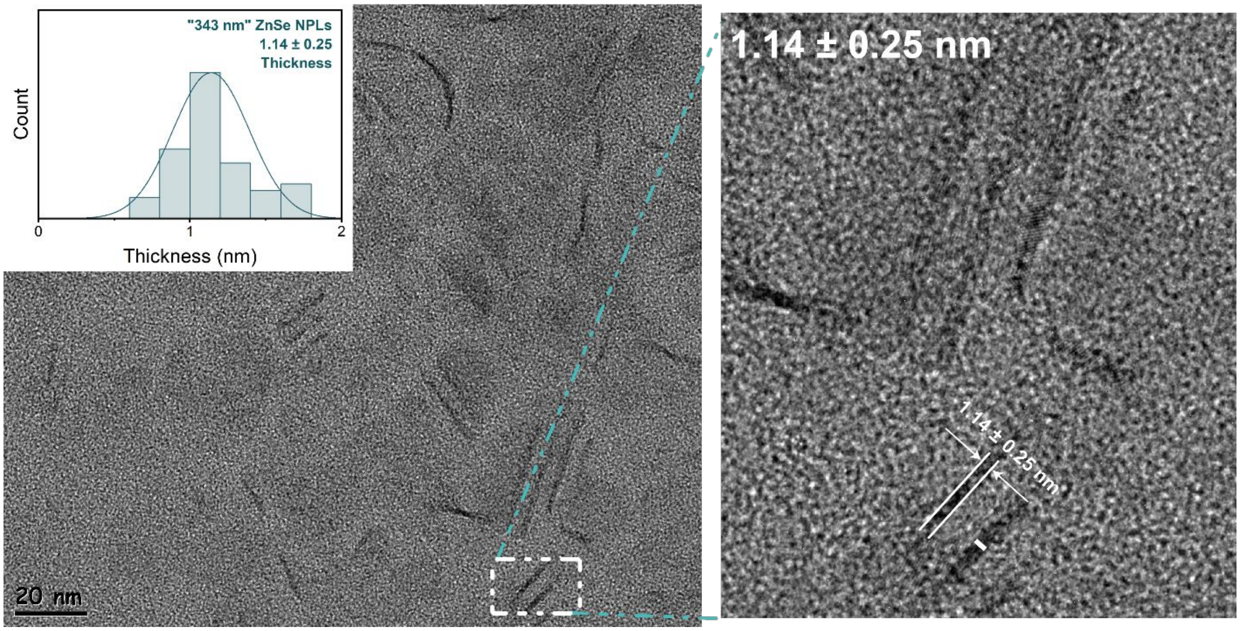


**Figure S5.** Transmission electron microscopy (TEM) image of "343 nm" ZnSe NPLs together with the size distribution histogram.

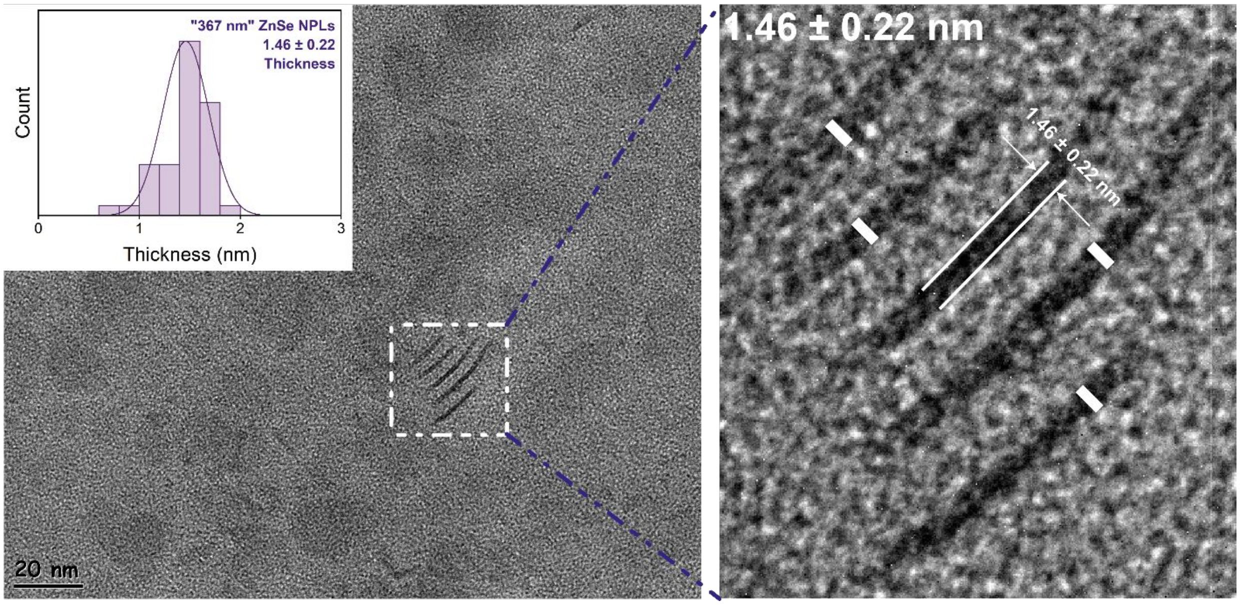


**Figure S6.** Transmission electron microscopy (TEM) image of "367 nm" ZnSe NPLs together with the size distribution histogram.

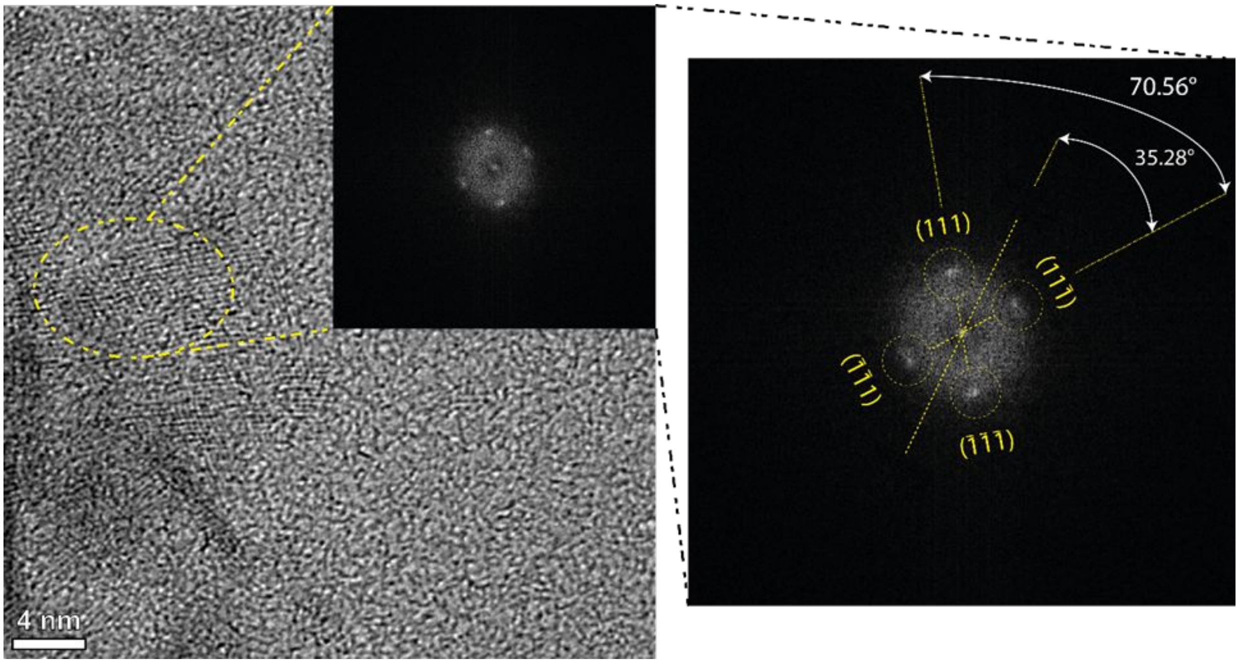


**Figure S7.** HRTEM image of ZnSe NPLs having a population of "343 nm" together with the FFT image of the selected are from the wide facet of ZnSe NPLs. The obtained FFT image confirms that the zone axis for the ZB ZnSe NPLs can be chosen as [110] direction.

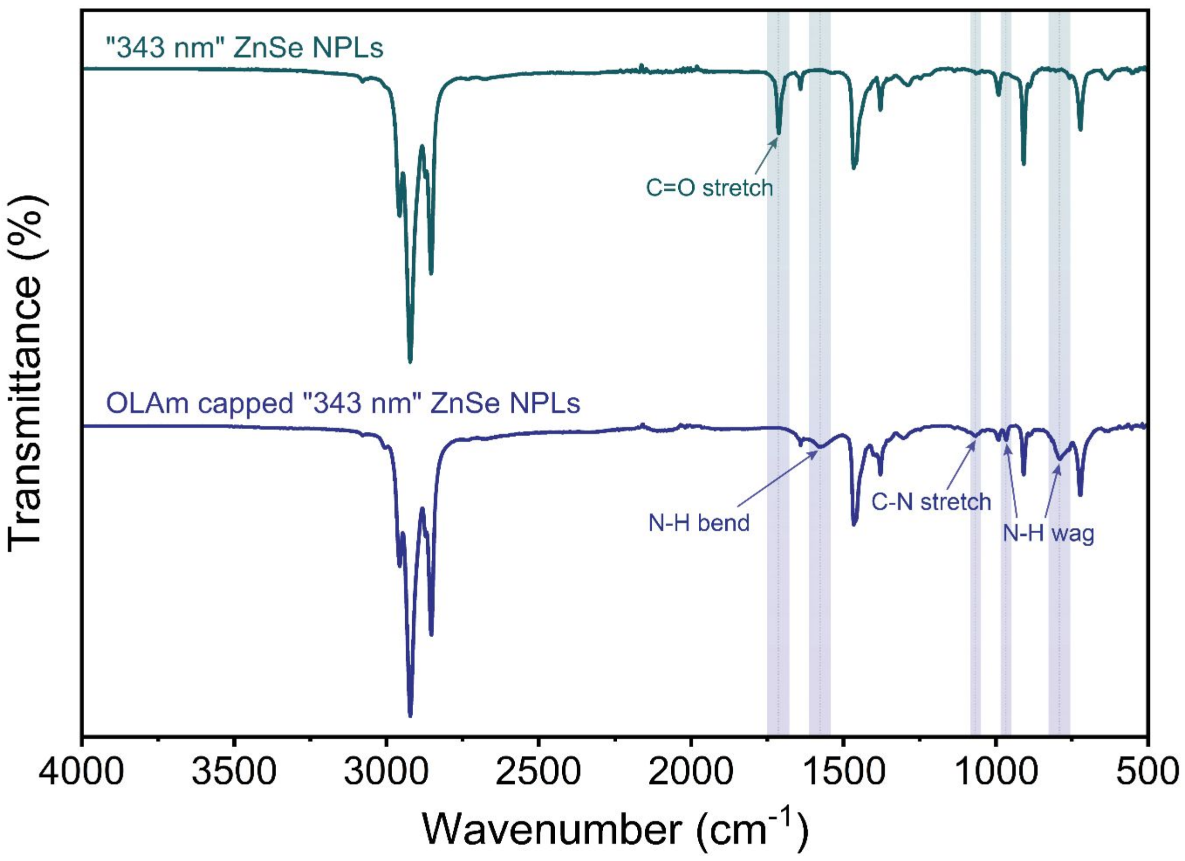


**Figure S8.** Fourier Transform Infrared (FTIR) spectra of ZB ZnSe NPLs having a population of "343 nm" before and after the ligand exchange.

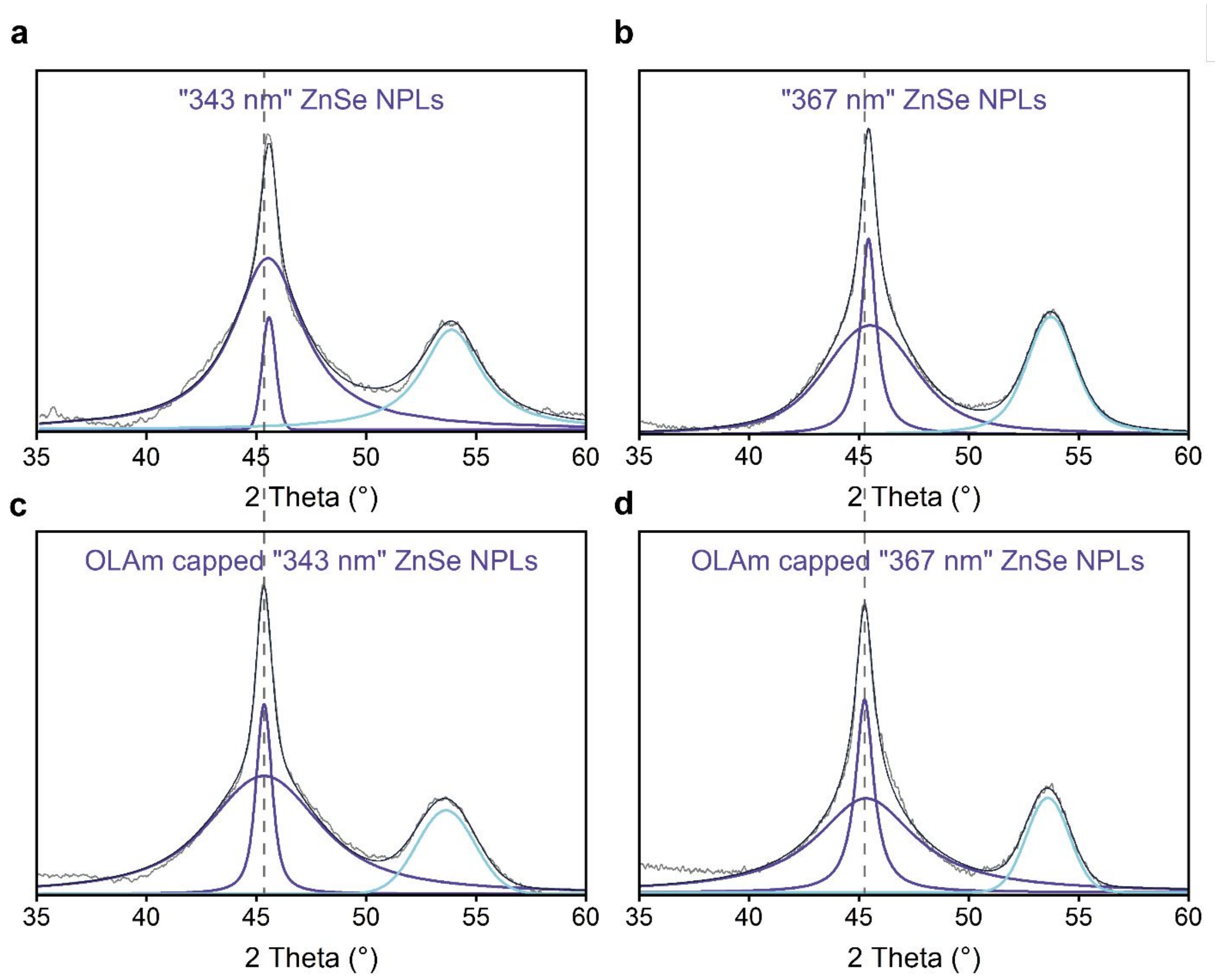


**Figure S9.** Fitting results of (220) and (311) peaks belonging to (a) "343 nm" ZnSe NPLs, (b) "367 nm" ZnSe NPLs, (c) OLAm capped "343 nm" ZnSe NPLs and (d) OLAm capped "367 nm" ZnSe NPLs. Grey curve – experimental data, black curve – cumulative curve, purple curve – fitted data of (220) peaks, and light blue – fitted data of (331) peaks.

**Table S1.** Interplanar distances calculated from the (220) diffraction peaks, lattice parameters, and unit cell volume of initial ZnSe NPLs and OLAm capped ZnSe NPLs.

| SAMPLE | $d_{220}$, (Å, *lateral)* | $d_{220}$, (Å, *thickness)* | a (Å) | c (Å) | V (Å$^3$) |
|---|---|---|---|---|---|
| **"343 nm"** ZnSe NPLs | 1.989 | 1.991 | 5.626 | **5.635** | 178.4 |
| OLAm capped **"343 nm"** ZnSe NPLs | 1.998 | 1.998 | 5.652 | **5.652** | 180.6 |
| **"367 nm"** ZnSe NPLs | 1.995 | 1.992 | 5.643 | **5.626** | 179.1 |
| OLAm capped **"367 nm"** ZnSe NPLs | 2.002 | 2.000 | 5.664 | **5.648** | 181.2 |
